# Long-term Survival and Prognostic Factors of Breast Cancer

**DOI:** 10.34172/aim.2022.96

**Published:** 2022-09-01

**Authors:** Asiie Olfatbakhsh, Leila Heidari, Zahra Omidi, Esmat-o-Sadat Hashemi, Maryam Ansari, Samaneh Mozaffarian, Shahpar Haghighat

**Affiliations:** ^1^Breast Cancer Research Center, Motamed Cancer Institute, ACECR, Tehran, Iran

**Keywords:** Breast cancer, Iran, Survival rate

## Abstract

**Background:**

Breast cancer survival rate is an important index for assessment of treatment effect in reducing the mortality. Weaimed to determine the fifteen-year survival rate for breast cancer at a referral center in Iran and its correlated factors.

**Methods:**

This survival study enrolled patients with breast cancer who referred to Motamed Cancer Institute (MCI) from 1998 to2016. The survival rate was calculated using the Kaplan-Meier method. The relationship of demographic, clinical and therapeuticfactors with overall survival (OS) was studied using Cox’s proportional hazard model.

**Results:**

Totally, 3443 patients were studied. Their mean age and follow-up period were 47.7 (±11.43) years and 61.66 (±52.1)months, respectively. The median follow-up time was 48.4 months (range: 1-413 months), 49.7% of the patients had high schoolor higher education, and 71.3% presented in the early stage of the disease. Death occurred in 505 (14.7%) of the patients. Theoverall 2-, 5-, 7-, 10- and 15-year survival rates were 93%, 82%, 78%, 74%, and 66%, respectively. Lymph node involvement(HR=2.07; 95% CI: 1.38–3.09), tumor size≥5 cm (HR=2.83; 95% CI: 1.59–2.04), being single/divorced/widowed (HR=1.65;95% CI: 1.13–2.4), and education level<high school diploma (HR=1.57; 95% CI: 1.13–2.17) were independent predictors ofbreast cancer survival.

**Conclusion:**

The five-year breast cancer survival rate in this study was higher than reported by some other studies in Iran, whichcould be due to the multidisciplinary treatment approach in MCI. Tumor size and lymph node involvement as indicators ofdelayed diagnosis may affect breast cancer survival, even though their true effect due to lead-time bias should be considered. Thecorrelation of education level with survival confirms the importance of awareness and the need to establish strategies for earlydiagnosis in Iranian women.

## Introduction

 Currently, cancers are considered an important health priority worldwide due to their chronic nature and burden, especially in developing countries. Cancers cause millions of deaths such that, in 2018, the cancer incidence and mortality worldwide were about 18 million and 9.5 million, respectively. Breast cancer is the most common cancer in women, diagnosed in about two million cases yearly. According to the Globocan report (2018), about 6 500 000 people died of breast cancer, accounting for 6.6% of all cancer deaths.^1^ The breast cancer incidence rate in Asia is 29 per 100 000 women, and it is increasing. In Iran, the breast cancer incidence rate is about 13000 cases which accounts for 11.95% of all cancers, and the ASR rate is 34.53 per 100 000 women.^2^

 Survival rate is necessary for assessing the clinical status and calculating the prognosis based on the disease features, treatment methods, and patients’ characteristics. Survival rates vary in different regions and are usually higher in developed countries because of screening and early detection strategies, high-quality surgery, and adjuvant therapies.^3^ Individual differences, healthcare system differences, public awareness about cancer, delayed diagnosis, disease staging, comorbidity, and optimal treatment availability are suggested as potential reasons for the differences in survival rates across countries.^4^ In the United States, the five-year survival rate is 89%, and the ten-year and fifteen-year survival rates are 84% and 80%, respectively.^5^ According to the Korea Central Cancer Registry report, the five-year and ten-year survival rates for all stages of breast cancer in 2014 were 91.2% and 84.8%, respectively.^6^ In Iran, several studies have reported survival rates in patients with breast cancer, especially five-year survival rate. A study reported a five-year survival rate of 62% in 163 patients with breast cancer.^7^ A national survey of 6147 breast cancer cases in 2010 reported the overall five-year survival rate at 71% in Iran.^8^ Most of the studies have estimated the survival rate in different distributions of public and private centers of the country, and 7-, 10-year, or longer rates have not been reported. Determining the survival rate helps healthcare providers design more effective and advanced treatment methods, improve disease control, and reduce the death rate.^8^ This study aimed to investigate the long-term survival rates and assess factors affecting survival rate in patients at a cancer referral institute.

## Material and Methods

 The present study recruited 3732 patients with breast cancer who referred to Motamed Cancer Institute (MCI), Tehran, Iran, from 1998 to 2016. Demographic characteristics (age at diagnosis, education level, marital status, and reproductive status), clinical variables (ER/PR, Her2, P53 receptors, type of tumor, type of surgery, tumor size, lymph node involvement status, stage and grade of the disease), and therapeutic data (chemotherapy, radiotherapy, and hormone therapy) were extracted from the patients’ records in the follow-up clinic and recorded in a checklist. In this clinic, all patients are followed every three months for the first two years and then every six months until the fifth year. After each visit, the results of the physical examination, lab tests, and symptoms are recorded. In cases with incomplete information, the patients were contacted for a telephone interview or an appointment. In cases without recorded data in the previous six months, the patient or their families were called to determine their latest status as alive, dead or censored. If someone refused to be followed up in the MCI clinic, necessary questions were asked, and if she was unwilling to be recruited in the study, her data was considered incomplete. Patients’ survival was considered as the time interval from diagnosis to death/last follow-up data. The first positive breast cancer pathology report was recorded as the diagnosis date.

###  Statistical Analysis

 Descriptive methods assessed demographic, clinical, and therapeutic variables. Death status was assigned as a binary variable (0 and 1). The Kaplan-Meier and Cox regression analysis proportionality assumptions were assessed by the log (-log) chart. Kaplan-Meier analysis and life tables estimated the probability of death. The correlation of demographic, clinical, and therapeutic factors with the survival rate was studied by Cox regression analysis.

## Results

 In total, 3443 patients were assessed. Patients were excluded if they presented to the follow-up clinic only once for one month after treatment, or did not answer our follow-up calls (n = 289). Because some patients’ records contained incomplete data for some variables, the cumulative frequency of most variables does not equal 3443. There were missing data in education level (5.3%), stage of disease (10.1%), grade of cancer (41.5%), estrogen receptor (ER) (38%), progesterone receptor (PR) (38.7%), Her2 (47.6%), P53 (74.7%), pathology report (14.3%), lymph nodes involvement (17.1%), tumor size (29.1%), type of surgery (16.6%), chemotherapy (22.9%), radiotherapy (29.2%), and hormone therapy (46%). Table 1 stratifies mean overall survival (OS) based on different demographic, clinical and therapeutic categories. Most patients were married and premenopausal, and had high school or higher education and a mean age of 47.7 ( ± 11.43) years. In terms of clinical characteristics, invasive ductal carcinoma, tumor size 2-5 cm, lymph node involvement, grade II and stage II of the disease were the most frequent categories. Chemotherapy, radiotherapy, hormone therapy and modified radical mastectomy (MRM) surgery were performed in 90.1%, 86.3%, 88.3%, and 55.1%, respectively. Most patients (72.3%) were diagnosed in the early stages, and most tumors were of grade II (59.7%).

**Table 1:** Demographic, Clinical, Therapeutic Characteristics and Overall Survival

**Variables**	**Patient Frequency**	**Death** **Frequency** ^*^	**Overall Survival (month)**
	**No. (%)**	**No. (%)**	**Mean (SE)**
**Demographic**			
Age at diagnosis (y)			
< 35	402 (11.7)	64 (15.9)	281 (17.99)
≥ 35	3041 (88.3)	441 (14.5)	266.03 (6.85)
< 40	850 (24.7)	119 (14)	298.43 (12.34)
≥ 40	2593 (75.3)	386 (14.9)	240.62 (6.31)
< 50	2078 (60.4)	269 (12.9)	291.74 (10.07)
≥ 50	1365 (39.6)	236 (17.3)	230.01 (6.94)
Education level			
< high school diploma	1640 (50.3)	291 (17.7)	266.43 (7.1)
≥ high school diploma	1619 (49.7)	188 (11.6)	307.65 (11.56)
Marital status			
Single/divorced/widowed	714 (20.7)	122 (17.1)	242.35 (13.28)
Married	2729 (79.3)	383 (14)	293.16 (8.6)
Reproductive status			
Menopausal	1377 (40)	234 (17)	258.66 (8.68)
Premenopausal	2066 (60)	271 (13.12)	298.04 (8.87)
**Clinical**			
Staging of breast cancer			
0	42 (1.4)	0 (0)	0 (0)
I	612 (19.8)	40 (6.5)	343.6 (14.1)
II	1595 (51.5)	186 (11.7)	276.6 (9.1)
III	697 (22.5)	162 (23.2)	164.1 (6.6)
IV	150 (4.8)	70 (46.7)	78.2 (5.6)
Grading of breast cancer			
I	231 (11.5)	30 (12.99)	174 (6.9)
II	1202 (59.7)	180 (14.97)	218.4 (6.9)
III	582 (28.9)	99 (17.01)	192.8 (7.6)
Estrogen receptor (ER)			
Negative	596 (27.9)	112 (18.79)	229.35 (9)
Positive	1539 (72.1)	236 (15.33)	215.1 (7)
Progesterone receptor (PR)			
Negative	739 (35)	140 (18.9)	228.6 (8.8)
Positive	1370 (65)	203 (14.8)	210.9 (8.2)
Her2 receptor			
Negative	1238 (68.6)	192 (15.5)	158.9 (3.5)
Positive	566 (31.4)	104 (18.4)	159.4 (5.6)
P53 receptor			
Negative	511 (58.6)	86 (16.8)	181.6 (4.8)
Positive	361 (41.4)	66 (18.3)	179.04 (4)
Pathology report			
*In situ* lobular carcinoma	22 (0.7)	1 (4.54)	112.5 (5.4)
*In situ* ductal carcinoma	128 (4.3)	4 (0.1)	194.16 (6.99)
Invasive ductal carcinoma	2478 (84)	398 (16.06)	230.06 (5.6)
Invasive lobular carcinoma	137 (4.6)	19 (13.9)	164.1 (8.1)
Others	185 (6.3)	21 (11.35)	315.4 (32.5)
Lymph nodes involvement			
Positive	1831(64.1)	352 (19.22)	243.39 (8.09)
Negative	1024 (35.9)	82 (8.01)	300.5 (11)
Tumor size (cm)			
< 2	646 (26.5)	43 (6.66)	277.75 (10.14)
2–5	1263 (51.8)	201 (15.91)	265.67 (9.32)
≥ 5	531 (21.8)	140 (26.37)	129.11 (6.82)
**Therapeutic**			
Type of surgery			
Modified radical mastectomy	1582 (55.1)	301 (19)	275.5 (8.6)
Breast preservation	1247 (34.4)	116 (9.3)	265.2 (10.16)
Bilateral	44 (1.5)	8 (18.2)	178 (19.9)
Chemotherapy			
No	262 (9.9)	27 (10.3)	328.7 (18.7)
Yes	2392 (90.1)	391 (16.3)	253.8 (8.8)
Radiotherapy			
No	334 (13.7)	55 (16.5)	312.9 (13.1)
Yes	2102 (86.3)	324 (15.4)	261.2 (8.2)
Hormone Therapy			
No	218 (11.7)	52 (23.9)	282.3 (16.7)
Yes	1642 (88.3)	235 (14.3)	277.3 (7.4)

^*^The frequency and percent of death have been calculated based on the number of subjects in each category.

 According to the Kaplan-Meier test, the highest frequency of death was observed in women aged 50 years and older (17.3%), with less than high school education (17.7%), of postmenopausal age (17%), and the single/divorced/widowed (17.1%). The frequency of death based on clinical variables was found to be 26.37% for tumor size ≥ 5 centimeters, 19.22% for positive lymph node involvement, 18.79% for negative ER, 18.9% for negative PR, 18.4% for positive Her2neu receptor, 18.3% for positive P53 receptor, 16.06% for invasive ductal carcinoma pathology, 46.7% for stage IV and 17.01% for grade III. In terms of treatment modalities, death rate was found to be 19% for MRM surgery, 16.3% for chemotherapy, 16.5% for not receiving radiotherapy, and 23.9% for not receiving hormone therapy (Table 1). Subgroup analysis showed that 87.4% of the patients in MRM group and 91.6% in the chemotherapy group were diagnosed in stages II, III, and IV of the disease. Not receiving radiotherapy increased the risk of death in the MRM group, as well (HR: 1.65, 95% CI: 1.2–2.26).

 The mean survival rate was higher in the following patients: age younger than 40 years (298.43), ≥ high school education (307.65), married (293.16), premenopausal (298.04), stage I (343.6), grade II (218.4), negative ER (229.35), negative PR (228.6), positive Her2 receptor (159.4), negative P53 receptor (181.6), pathology reports other than invasive ductal carcinoma (315.4), no lymph node involvement (300.5), tumor size < 2 cm (277.75), MRM surgery (275.5), and patients not receiving chemotherapy (328.7), radiotherapy (312.9), or hormone therapy (282.3) (Table 1). Stratified analysis showed the five-year survival rate of patients < 40 years at 76% ( ± 0.023) while it was 84% ( ± 0.009) in patients ≥ 40 years. The relative frequency of ER and PR negative categories in primary breast cancers (Stage I and II) was 54.4% and 53.4%, respectively.

**Figure 1:**
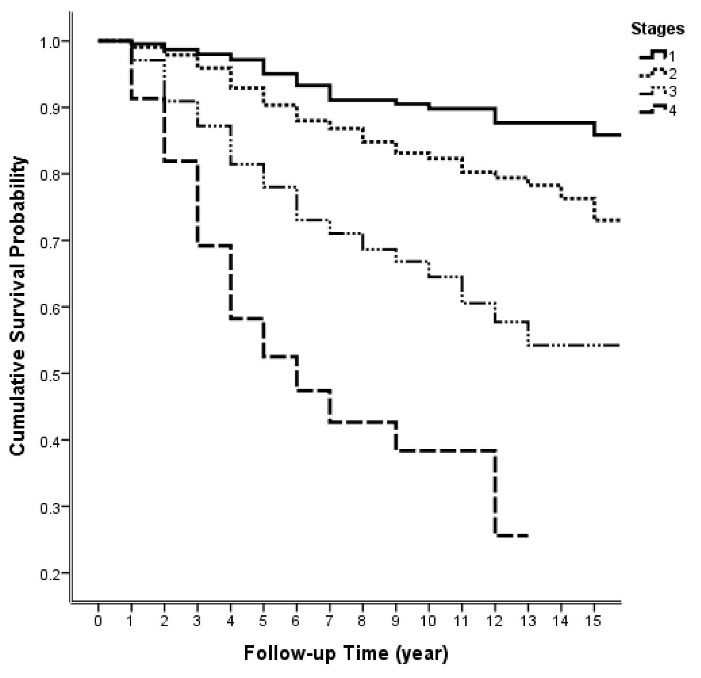
Survival Probability Trend During Fifteen Years of Follow-up in Different Stages of the Disease

 The mean follow-up period was 61.66 ( ± 52.1) months: 21.7% were followed less than two years, 38.3% more than five years, and 12.7% more than ten years. As shown in Table 2, 498 deaths occurred over the fifteen years of follow-up. According to the life tables results, the five-year and ten-year survival rates were estimated to be 82% and 74%, respectively. The first quartile of survival time was 120 (95% CI: 103.5-136.5) months. Figure 1 demonstrates the two-, five-, seven-, ten-, and fifteen-year survival rates in different stages.

**Table 2:** The Cumulative Probability of Survival Over 15 Years in Breast Cancer Patients (n = 3443)

**Time Interval**	**Number of Patients at the End of the Period**	**Cumulative Frequency of Deaths**	**Probability of Survival (SE)**
Two-year	2698	148	0.93 (0.002)
Five-year	1319	366	0.82 (0.01)
Seven-year	793	432	0.78 (0.01)
Ten-year	436	468	0.74 (0.01)
Fifteen-year	129	498	0.66 (0.02)

 The Cox regression model analyzed the correlation of prognostic factors with survival rate (Table 3). For each factor, the reference category was indicated as HR equal to one.

**Table 3:** Cox Analysis to Assess the Relationship Between Demographic, Clinical and Therapeutic Variables with the Survival of the Participants (n = 3443)

**Predictors Variable**	**Number of Patients**	**Number of Death (%)**	**Hazard Ratio (95% CI)**	
			**Univariate**	**Multivariate**
Age (y)				
< 50	2078	269 (12.9)	1	-
≥ 50	1365	236 (17.3)	1.45 (1.22–1.73)^*^	-
Education level				
≥ high school diploma	1619	188 (11.6)	1	1
< high school diploma	1640	291 (17.7)	1.8 (1.5–2.16)^*^	1.57 (1.13–2.17)^*^
Marital status				
Married	2729	383 (14)	1	1
Single/divorced/ widowed	714	122 (17.1)	1.28 (1.05–1.57)^*^	1.65 (1.13–2.4)^*^
Reproductive status				
Menopausal	2066	234 (17)	1	**-**
Premenopausal	1377	271 (13.12)	0.83 (0.7–0.99)^*^	-
Lymph node involvement				
Negative	1024	82 (8.01)	1	1
Positive	1834	352 (19.22)	3.03 (2.38–3.85)^*^	2.07 (1.38–3.09)^*^
Tumor size (cm)				
< 2	646	43 (6.66)	1	1
2–5	1263	201 (15.91)	2.37 (1.7–3.3)^*^	1.52 (0.89–2.59)
≥ 5	531	140 (26.37)	5.7 (4.04–8.03)^*^	2.83 (1.59–2.04)^*^
Type of surgery				
Breast preservation	1247	116 (9.3)	1	**-**
MRM	1582	301 (19)	1.84 (1.49–2.29)^*^	-
Pathology Report				
Others	472	45 (9.53)	1	**-**
Invasive ductal carcinoma	2478	398 (16.06)	1.58 (1.16–2.16)^*^	-
Grading of disease				
I	231	30 (12.99)	1	**-**
II	1202	180 (14.97)	1.17 (0.8–1.73)	-
III	582	99 (17.01)	1.55 (1.03–2.23)^*^	**-**
Estrogen receptor				
Positive	1539	236 (15.33)	1	**-**
Negative	596	112 (18.79)	1.23 (0.98–1.53)	-
Her2 receptor				
Negative	1238	192 (15.5)	1	**-**
Others	566	104 (18.4)	1.21 (0.95–1.54)	-
Chemotherapy				
Yes	2392	391 (16.3)	1	**-**
No	262	27 (10.3)	0.53 (0.36–0.78)^*^	-
Radiotherapy				
Yes	2102	324 (15.4)	1	**-**
No	334	55 (16.5)	0.85 (0.64–1.13)	-
Hormone therapy				
Yes	1642	235 (14.3)	1	**-**
No	218	52 (23.9)	1.48 (1.09–1.99)^*^	-

^*^Significant statistical correlation (*P* < 0.05).

 The univariate Cox regression analysis showed that survival rate correlated significantly with age ≥ 50 years (HR = 1.45; 0.95 CI: 1.22–1.73), less than high school education (HR = 1.8; 0.95 CI: 1.5–2.16), being married (HR = 1.28; 0.95 CI: 1.05–1.57), premenopausal status (HR = 0.83; 0.95 CI: 0.7–0.99), positive lymph node involvement (HR = 3.03; 0.95 CI: 2.38–3.85), tumor size of 2-5 cm (HR = 2.37 0.95 CI: 1.7–3.3) and ≥ 5 cm (HR = 5.7; 0.95 CI: 4.04–8.03), stages II (HR = 1.8; 0.95 CI: 1.28-2.54), III (HR = 4.4; 0.95 CI: 3.11–6.22), and IV of the disease (HR = 11.22; 0.95 CI: 7.59–16.58), MRM type of surgery (HR = 1.84; 0.95 CI: 1.49–2.29), invasive ductal carcinoma (HR = 1.58; 0.95 CI: 1.16–2.16), grade III (HR = 1.55; 0.95 CI: 1.03–2.23), not receiving chemotherapy (HR = 0.53; 0.95 CI: 0.36–0.78), and not receiving hormone therapy (HR: 1.48; 0.95 CI: 1.09–1.99) (Table 3).

**Figure 2:**
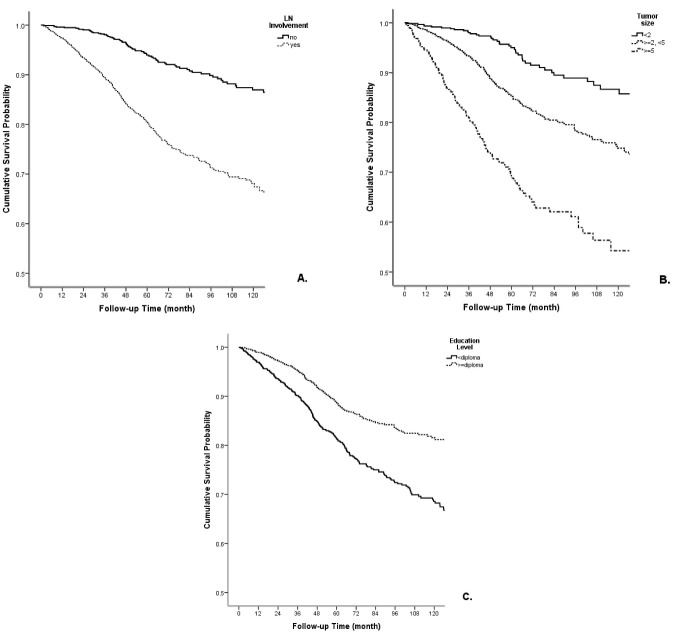
The Correlation of A) Lymph Node Involvement, B) Tumor Size, and C) Education with the Cumulative Survival Rate

 The interaction effect of the demographic and clinical variables with survival rate was assessed by the multivariate Cox regression forward method (Table 3). Stage of disease was not included in multivariate analysis because of its collinearity with tumor size and lymph node status. Lymph node involvement (HR = 2.07; 0.95 CI: 1.38-3.09), less than high school education (HR = 1.57; 0.95 CI: 1.13–2.17), tumor size of ≥ 5 cm (HR = 2.83; 0.95 CI: 1.59-2.04), and being single/divorced/widowed (HR = 1.65; 0.95 CI: 1.13–2.4) showed significant associations with lower survival rate (Figure 2).

## Discussion

 Overall, this study showed that the 2-, 5-, 7-, 10-, and 15-year survival rates in MCI were 93%, 82%, 78%, 74% and 66%, respectively. Lymph nodes involvement, less than high school education, and tumor size of ≥ 5 cm showed significant associations with lower survival rate.

 The study population’s mean age was 47.7 ( ± 11.43) years, and 60% of them were younger than 50 years. Another study at MCI in 2013 reported the mean age of patients at 46.5 ( ± 11.2) years.^9^ A meta-analysis including 24 survival studies from Iran also reported the mean age of the patients at 48.27 (CI = 43.68–52.86) years,^10^ which indicates an increasing trend in breast cancer diagnosis age. In most Iranian studies, like other Asian countries, breast cancer’s median age is under 50 years,^11-16^ which is different from the median age of the Western countries that are mostly above 50.^4,17^

 Our findings indicated that the five-year survival rate was 76% ( ± 0.023) for patients < 40 years and 84% ( ± 0.009) for patients ≥ 40 years. Younger patients usually present in a later stage, have negative ER and receive more aggressive treatments. In most studies, patients aged < 35 years have worse OS and recurrence-free survival. For example, in one study, the five-year survival rate for women < 35 years was 75% versus 84%–88% in women aged 35 to 69 years.^18,19^ The higher OS of this group in our study is related to their distribution in ER-negative groups or different stage groups, which means that the OS of patients < 40 was higher; however, it was worse in higher stages. Unrelated to the stage and tumor subtype, the health status of younger patients is better, and the OS of patients with good prognostic tumor characteristics could be better.

 In this study, we noticed a paradoxical result regarding the correlation of ER status, mean of survival months and the frequency of deaths. Even though death frequency was higher in ER-negative (18.8%) than ER-positive (15.3%) tumors, the mean of survival months was slightly higher in ER-negative groups. It may be related to the 38% missing data of ER status, and its interpretation requires caution.

 The results showed that the breast cancer death rate in single/divorced/widowed women was higher than married patients (17.1% vs 14%). Some studies have demonstrated that married patients with breast cancer are less likely to be diagnosed with a high stage of the disease or die of cancer.^20-22^ This finding may be partly attributed to breast density in younger unmarried women. Aizer and colleagues showed that survival benefits from marriage are even greater than chemotherapy. Distress and depression are more common in unmarried women (single/divorced/widowed), mediating poorer adherence to prescribed treatment and poorer survival.^23^ It can be concluded that married patients’ psychosocial support may play an important role in their higher life expectancy. It appears that establishing social support networks may be beneficial in improving the survival of unmarried patients.

 In terms of reproductive status, 60% of our patients were menopausal. A population-based study in 41 countries showed that low- and middle-income countries suffered higher incidence and mortality rates of breast cancer in premenopausal patients than high-income countries (55.2% vs 20.7%). Heer et al showed that the age-standardized incidence rates of breast cancer for postmenopausal cases are higher in developing countries. It appears that factors such as low physical activity, changes in lifestyle and reproductive behaviors and early menarche should be investigated further.^24^

 In this study, 71.3% of patients were diagnosed in early stages (I, II) and 27.3% in advanced stages (III, IV). The frequency of early-stage breast cancer in different countries such as Japan, Hong Kong, Korea, China, and Egypt has been reported at 89%, 82%, 81%, 74%, and 66%, respectively.^25^ On the other hand, 66% of cases in the United States are diagnosed in stage I, 26% in stage II/ III, and 5% in stage IV. This disparity appears to be mainly related to the screening mammography programs.^26^ As shown in the results, the early-stage diagnosis rate in the current study is similar to most developing countries. The stage at admission is mostly related to the patients’ socio-economic level and access to the health system.^25^

 Death was more frequent in patients who did not receive radiotherapy. As many MRM cases do not receive radiotherapy, we performed a subgroup analysis in the MRM group. The results showed that failure to receive radiotherapy increased the risk of death in the MRM group (HR: 1.65, 95% CI: 1.2–2.26). Post-mastectomy radiotherapy (PMRT) has two potential benefits: decreased locoregional recurrence rate and increased long-term breast cancer-specific and OS for specific patient populations. These benefits have been consistently reported in multiple studies.^27-29^ The most important indications for PMRT are LN involvement, T4 disease, positive margins and poor prognostic features (age ≤ 50 years, triple-negative histology, high grade, or lymphovascular invasion). The worse survival in patients who underwent MRM but did not receive radiotherapy is compatible with previous results. It may be related to receiving out-of-protocol treatment or being referred to other centers. Contrary to radiotherapy, a higher death rate was recorded in patients receiving chemotherapy, which could be due to the higher stages of the disease.

 The 2-, 5-, 7-, 10-, and 15-year survival rates were 93%, 82%, 78%, 74%, and 66%, respectively. The five-year survival rate of breast cancer in Iranian women was reported at 71% by the population-based cancer registry of the Ministry of Health in 2015.^2^ According to SEER data, American women’s five-year survival rate was 91% in 2020.^30^ The Japanese, Korean, Turkish, and Arab females’ survival rates were 88.1%, 83.7%, 76.7%, and 64.5%, respectively, before 2000.^25^ Comparing the five-year survival rate of this single-center (82%) to the similar mentioned studies reveals that the five-year survival rate in Iran is somewhat higher than many Asian and other developing countries. Using an integrated treatment and diagnosis guideline, which is updated every 2–3 years, and the breast cancer specialized team of MCI may explain why this center has a higher survival rate.

 The current study’s two- and five-year survival rates were 93% and 82%, respectively, similar to previous studies in MCI on 623 cases in 2013.^9^ Other studies in Iran have reported 5-, 7-, 10-, and 15-year breast cancer survival rates of about 45%–92%, 54.8%–76%, 31%–77%, and 46%, respectively.^10,31,32^ Similarly, in the past decade, patients’ survival rates improved from 85% to 90% in the USA and from 60% to 74% in Eastern Europe.^31^ Although our results indicate improvements in the breast cancer survival rate in Iran, it should be considered that MCI is a semi-public center with a multidisciplinary treatment protocol. So, this may not be generalized to all cancer centers in Iran. Besides, the results should be interpreted with caution due to the heterogeneity of studies in Iran compared to other countries regarding sample size, study population, etc.

 A significant association was observed between survival rate reduction and the variables of patients’ education level, lymph node involvement, single/divorced/widowed marital status, and tumor size ≥ 5cm. The correlations of age,^31,33,34^ lymph node involvement, tumor size,^31,35^ type of surgery, BMI,^34^ stage, and grade of the disease^33^ with breast cancer survival rate have been presented in previous Iranian studies.

 The relationship of education status with breast cancer survival may be due to low awareness, lower socioeconomic status, living in rural areas with poor access to health centers, and delay in breast cancer diagnosis. The patient delay can lead to breast cancer detection at higher stages with more involved lymph nodes and larger tumor size, highlighting the importance of early detection policies in improving breast cancer survival rate. In some Asian countries such as Singapore, Taiwan, and Korea, where screening has been promoted, relatively reasonable survival rates have been reported.^25^ Early detection can definitely improve breast cancer survival rate, but it is difficult to estimate its actual effect due to lead-time bias. In different countries, the survival rate should be interpreted with caution by considering this bias effect. As the incidence of breast cancer increases in Asia, health policymakers and future studies should attend to improving awareness, arranging comprehensive and population-based screening programs, providing easy access to the physical exam and health services, enhancing cancer registration system, running epidemiologic studies, and progress in treatment methods.

 In conclusion, the survival rate in this study was higher than previous Iranian studies and comparable to many developed countries. This higher survival rate can be related to our multidisciplinary protocol-based treatment and improved diagnostic methods. Prognostic factors of survival rate such as lower education, being unmarried, lymph node involvement and tumor size > 5 cm emphasize the importance of health education programs and women’s awareness about breast cancer and its early detection.
